# RETRACTION: Utilizing the Visual Analogue Scale (VAS) to Monitor and Manage Pain in Post‐Operative Skin Wounds After Thoracic Surgery

**DOI:** 10.1111/iwj.70471

**Published:** 2025-04-02

## Abstract

**RETRACTION:** QuM. , ZhaoJ. , ZhangY. , XuZ. , MaC. , and CuiH. , “Utilizing the Visual Analogue Scale (VAS) to Monitor and Manage Pain in Post‐Operative Skin Wounds After Thoracic Surgery,” International Wound Journal 21, no. 3 (2024): e14503, 10.1111/iwj.14503.37969025 PMC10898399

The above article, published online on 15 November 2023, in Wiley Online Library (http://onlinelibrary.wiley.com/), has been retracted by agreement between the journal Editor in Chief, Professor Keith Harding; and John Wiley & Sons Ltd. Following an investigation by the publisher, all parties have concluded that this article was accepted solely on the basis of a compromised peer review process. The editors have therefore decided to retract the article. The authors did not respond to our notice regarding the retraction.

**RETRACTION:** M. Qu , J. Zhao , Y. Zhang , Z. Xu , C. Ma , and H. Cui , “Utilizing the Visual Analogue Scale (VAS) to Monitor and Manage Pain in Post‐Operative Skin Wounds After Thoracic Surgery,” International Wound Journal 21, no. 3 (2024): e14503, 10.1111/iwj.14503.37969025 PMC10898399

The above article, published online on 15 November 2023, in Wiley Online Library (http://onlinelibrary.wiley.com/), has been retracted by agreement between the journal Editor in Chief, Professor Keith Harding; and John Wiley & Sons Ltd. Following an investigation by the publisher, all parties have concluded that this article was accepted solely on the basis of a compromised peer review process. The editors have therefore decided to retract the article. The authors did not respond to our notice regarding the retraction.

